# Characterization and stress-responsive regulation of CmPHT1 genes involved in phosphate uptake and transport in Melon (*Cucumis melo* L.)

**DOI:** 10.1186/s12870-024-05405-w

**Published:** 2024-07-23

**Authors:** Pengli Li, Asad Rehman, Jing Yu, Jinyang Weng, Beibei Zhan, Yueyue Wu, Yidong Zhang, Liying Chang, Qingliang Niu

**Affiliations:** 1 Key Laboratory of Urban Agriculture (South), Ministry of Agriculture, School of Agriculture and Biology, Shanghai Jiao Tong University, Shanghai, 200240 China; 2 Jiangsu Agri-animal Husbandry Vocational College, Taizhou, China

**Keywords:** Phosphate transporter, Expression pattern, Melon, Stress, WRKY transcription factor

## Abstract

**Background:**

Phosphorus (P) deficiency, a major nutrient stress, greatly hinders plant growth. Phosphate (Pi) uptake in plant roots relies on PHT1 family transporters. However, melon (*Cucumis melo* L.) lacks comprehensive identification and characterization of PHT1 genes, particularly their response patterns under diverse stresses.

**Results:**

This study identified and analyzed seven putative *CmPHT1* genes on chromosomes 3, 4, 5, 6, and 7 using the melon genome. Phylogenetic analysis revealed shared motifs, domain compositions, and evolutionary relationships among genes with close histories. Exon number varied from 1 to 3. Collinearity analysis suggested segmental and tandem duplications as the primary mechanisms for *CmPHT1* gene family expansion. *CmPHT1;4* and *CmPHT1;5* emerged as a tandemly duplicated pair. Analysis of cis-elements in *CmPHT1* promoters identified 14 functional categories, including putative PHR1-binding sites (P1BS) in *CmPHT1;4*, *CmPHT1;6*, and *CmPHT1;7*. We identified that three WRKY transcription factors regulated *CmPHT1;5* expression by binding to its W-box element. Notably, *CmPHT1* promoters harbored cis-elements responsive to hormones and abiotic factors. Different stresses regulated *CmPHT1* expression differently, suggesting that the adjusted expression patterns might contribute to plant adaptation.

**Conclusions:**

This study unveils the characteristics, evolutionary diversity, and stress responsiveness of *CmPHT1* genes in melon. These findings lay the foundation for in-depth investigations into their functional mechanisms in Cucurbitaceae crops.

**Supplementary Information:**

The online version contains supplementary material available at 10.1186/s12870-024-05405-w.

## Background

Phosphorus (P) is an essential element of phospholipids and nucleic acids, which plays a crucial role in energy transfer reactions and signal transduction processes that are vital for all life forms on Earth [1]. Although soils generally contain abundant P, only a small fraction is accessible for crop use [2]. It is bound to incompletely weathered mineral particles, adsorbed on mineral surfaces, or forms occluded P through secondary mineralization [2]. Therefore, roots obtain phosphate (Pi) against a huge concentration gap between plant cells (mM) and soil (µM) [3, 4]. So, efficient Pi acquisition from soil and translocation within plants are necessary to maintain general levels of cellular Pi [5]. Root cell absorption of Pi from the soil is energy-dependent. The PHT1 family of plant Pi transporters are known to be the primary facilitator of this process [6, 7].

The first Pi transporters (PTs) identified were AtPHT1;1 and AtPHT1;4 in higher plant [7]. So far, 9 *AtPHT1s* have been found in Arabidopsis thaliana [8]. 13 *OsPHT1s* have been discovered in rice (*Oryza sativa*) [9]. These PHT1s have distinct functions in the absorption, movement, and storage of Pi. In Arabidopsis, disruption of *AtPHT1;1* and *AtPHT1;4* leads to Pi uptake to reduce to 57% of the wild-type under Pi deficiency, and 70% of the wild‐type under Pi sufficiency [10]. *AtPHT1;8* and *AtPHT1;9* mediate inorganic phosphate acquisition during Pi starvation [8]. In rice, mutation of *OsPHT1;2*, *OsPHT1;3*, *OsPHT1;6*, or *OsPHT1;8* reduces Pi uptake under low Pi regimes [9, 11, 12], and mutation of *OsPHT1;1*, *OsPHT1;4*, or *OsPHT1;8* reduces Pi uptake under Pi sufficiency [11, 13, 14]. *OsPHT1;9* and *OsPHT1;10* redundantly function in Pi uptake under both high- and low-Pi conditions [15]. *GhPHT1;4* and *GhPHT1;5* were responsible for Pi uptake under Pi-starvation conditions in cotton [16]. The transgenic plants overexpressing *MtPT5* in *M. truncatula* showed larger leaves, higher biomass and Pi enrichment compared with wild type [17]. Expression of *PnPht1;1* or *PnPht1;2* in mutant strains could enhance the uptake of Pi in *Panax notoginseng* [18]. Collectively, these studies confirm that PHT1 proteins play important roles in Pi acquisition from the rhizosphere into plant roots under different Pi supply.

Commonly, *PHT1* family members are highly conserved among the different species [19]. 36 *TaPHT1s* have been found in wheat (*Triticum aestivum*), among which *TaPHT1.1/1.9*, *1.2*, and *1.10* were expressed especially in roots [20]. There are 14 *PHT1*s in soybean (*Glycine max* (L.) Merr.), which are expressed differently not only in responding to Pi availability but also in other nutrient deficiencies, including N, K, and Fe, in the different tissues [21]. 8 *PHT1*s showed diverse roles and genetic redundancy responding to Pi deficiency in tomato [22]. Genome-wide analysis has identified 12 *PHT1*s in *Gossypium hirsutum* [23]. 9 *CsPHT1s* were identified in tea plants (*Camellia sinensis* L. O. kuntze), which play crucial roles in selenite homeostasis [24]. 201 *PHT1* homologs were identified and analyzed from three diploids and two allotetraploids Brassica species, which were induced by heavy metal stress [25]. However, the characterization of PHT1 gene family and the stress-responsive patterns are still unknown in melon.

Melon is an important crop for its unique flavor and nutritional value worldwide [26]. Pi uptake and transport mediated by PHT1 play an important role in melon plant growth, fruit growth and quality formation, especially sugar accumulation. Furthermore, current melon cultivars have a narrow genetic base and are vulnerable to both biotic and abiotic stresses due to prolonged domestication and artificial selection for high yields and desirable traits [27, 28]. Therefore, breeders are facing the arduous work of improving melon resistance with conventional and/or modern breeding approaches. Our previous research revealed that three *CmPHT1s* positively participated in Pi uptake under Pi-deficiency [29]. We speculated that there should be other *PHT1s* in melon. Are *CmPHT1s* involved in the other stress responses in melon? Further, what factors regulate the transcription of these *PHT1* genes? In the current study, 7 *CmPHT1* genes were identified and systematically analyzed based on the updated melon genome and their characteristics were analyzed including gene structure, conversed motif structure, chromosomal location, evolutionary relationship, synteny relationship, and cis-elements in the promoters. 14 cis-element categories were identified in *CmPHT1* promoters. The stress-responsive expression patterns of *CmPHT1s* in melon were surveyed under low-phosphate stress (LP) and other stresses. Combining the bioinformatical analysis, three CmWRKYs regulated CmPHT1;5 expression by binding to its W-box element. This research shed light on the characterization and stress-regulatory elements of *CmPHT1s*, which contribute to clarify further the functional mechanisms under biotic and abiotic stress in melon.

## Results

### 7 *CmPHT1s* were identified in melon

The BLAST search was used to identify all possible *PHT1* members in melon genome. After the redundant and disrelated genes were deleted, and the conserved domains were ensured, 7 *CmPHT1s* were identified (Table S1). They were named *CmPHT1;1* to *CmPHT1;7* according to their chromosomal location. The CmPHT1 protein sequences exhibited high identity and similarity levels (Table S2). The identity of protein sequences ranged from 47 to 80%. The similarities ranged from 62 to 89%. The highest identity and similarity were found between the protein sequences of *CmPHT1;3* and *CmPHT1;5.*

The basic characteristics were analyzed (Table S1). These CmPHT1s ranged from 519 (CmPHT1;4) to 556 (CmPHT1;7) amino acids in size, with a molecular weight of around 56 to 61 kDa. PI values were from 8.57 (CmPHT1;4) to 9.07 (CmPHT1;1). The proteins with II value over 40 are unstable and those below 40 are stable proteins [30]. All CmPHT1 proteins are stable proteins. AI of 7 CmPHT1 proteins ranged from 88.08 (CmPHT1;2) to 94.68 (CmPHT1;5). The sub-cellular localization of the CmPHT1s was predicted using protein localization prediction software. The consistent results revealed that all CmPHT1 proteins were localized in the plasma membrane. These transporters shared a similar topology with 12 membrane-spanning domains.

### Analysis of gene structure of CmPHT1s

**Fig. 1:**
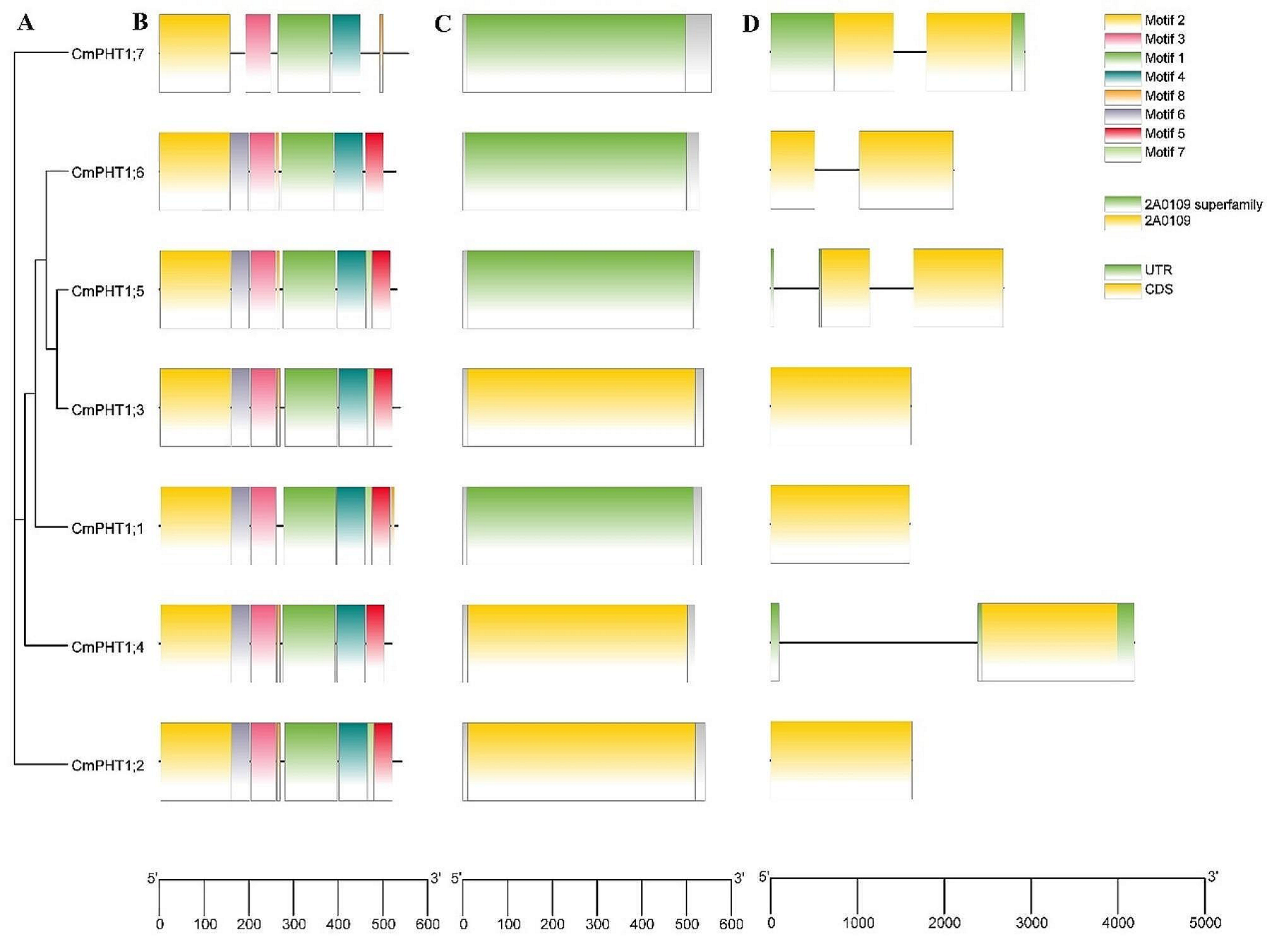
The phylogenetic tree, conserved protein motifs, conserved domain, and gene structure of *CmPHT1s*. (**A**) the phylogenetic tree; (**B**) conserved motifs in the CmPHT1 proteins. Each motif was represented by a colored box. (**C**) conserved domain; (**D**) gene structures of *CmPHT1s*. A yellow box, the black lines, and a green box represented the exons, introns, and UTR, respectively

To categorize the *CmPHT1* genes, we constructed a phylogenetic tree based on the protein sequences of CmHT1s (Fig. 1A). 8 conserved motifs, named Motif 1-Motif 8, were identified in CmPHT1s with MEME program (Fig. 1B and Table S3). CmPHT1;3 and CmPHT1;5 shared the same motifs, corresponding to the identity and similarity (Table S2). The conserved domains of *CmPHT1s* belonged to the 2A0109 superfamily and 2A0109 (Fig. 1C and Table S3). The superfamily is the phosphate: H^+^ symporter. *CmPHT1;1*, *CmPHT1;2*, *CmPHT1;3*, and *CmPHT1;4* had one exon. *CmPHT1;5*, *CmPHT1;6*, and *CmPHT1;7* had two exons (Fig. 1D and Table S3).

### Phylogenetic analysis of the CmPHT1 proteins

**Fig. 2:**
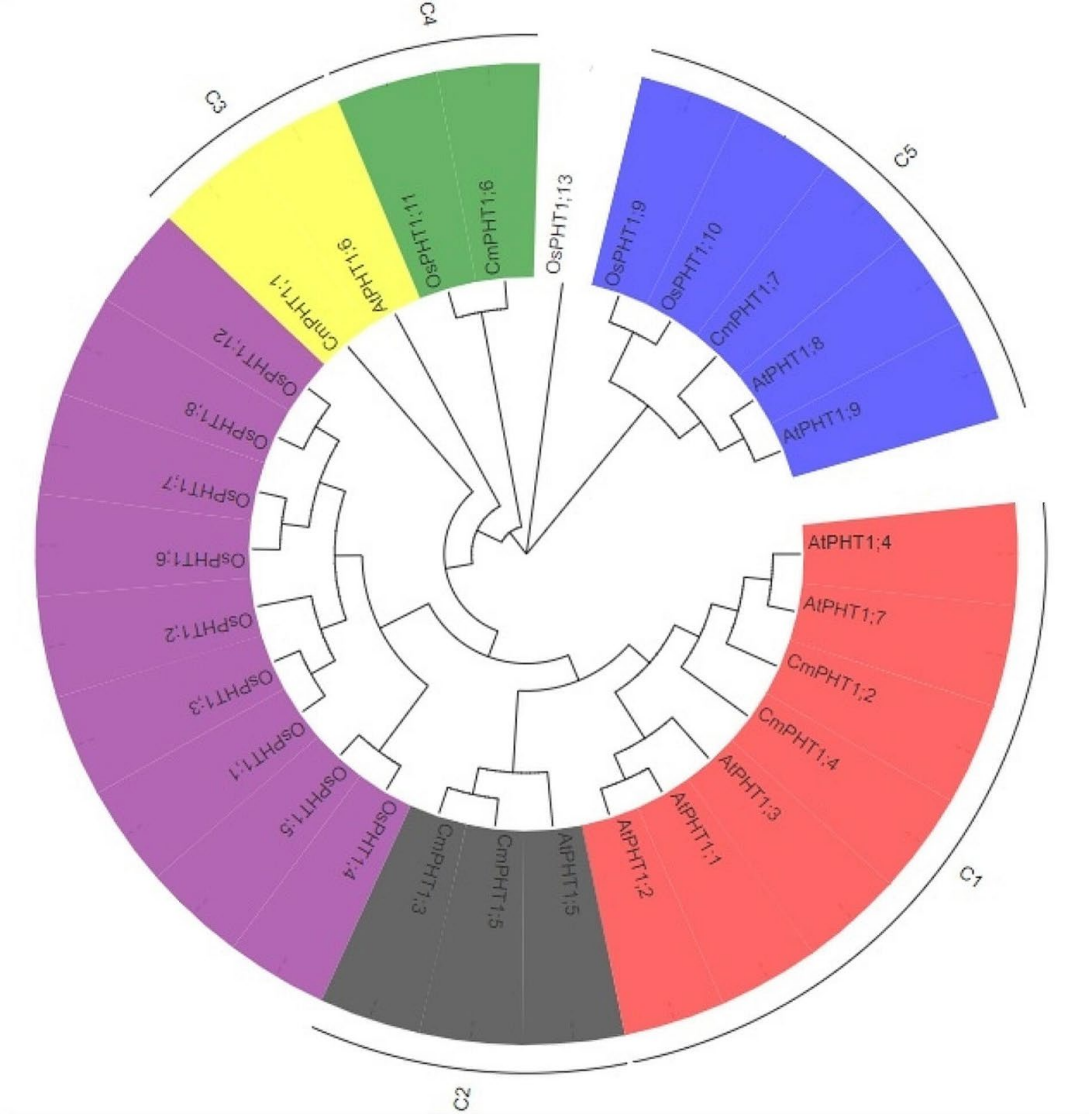
Evolutionary relationships of PHT1 proteins between melon, *Arabidopsis*, and rice. The tree was generated with the maximum likelihood method with 1000 bootstrap replicates based on multiple alignments of amino acid sequences of 7 CmPHT1s, 9 AtPHT1s, and 13 OsPHT1s. The different colored backgrounds represented clusters of CmPHT1s.

To predict the phylogenetic relationship and function of CmPHT1 proteins, a phylogenetic tree was constructed using the full-length PHT1 protein sequences of melon, *Arabidopsis*, and rice (Fig. 2). The CmPHT1s were clustered into five clusters (C1, C2, C3, C4, and C5). The phylogenetic analysis revealed that each gene from melon exhibited the closest relationship with corresponding genes from Arabidopsis. The following pairs were yielded: *CmPHT1;2*/*1;4* and *AtPHT1;4*, *CmPHT1;3*/*1;5* and *AtPHT1;5*, *CmPHT1;1* and *AtPHT1;6*, *CmPHT1;7* and *AtPHT1;8*, *CmPHT1;6* and *OsPHT1;11*. The results revealed the homologous relations among species.

### Distribution and expansion pattern analysis of CmPHT1 genes

**Fig. 3:**
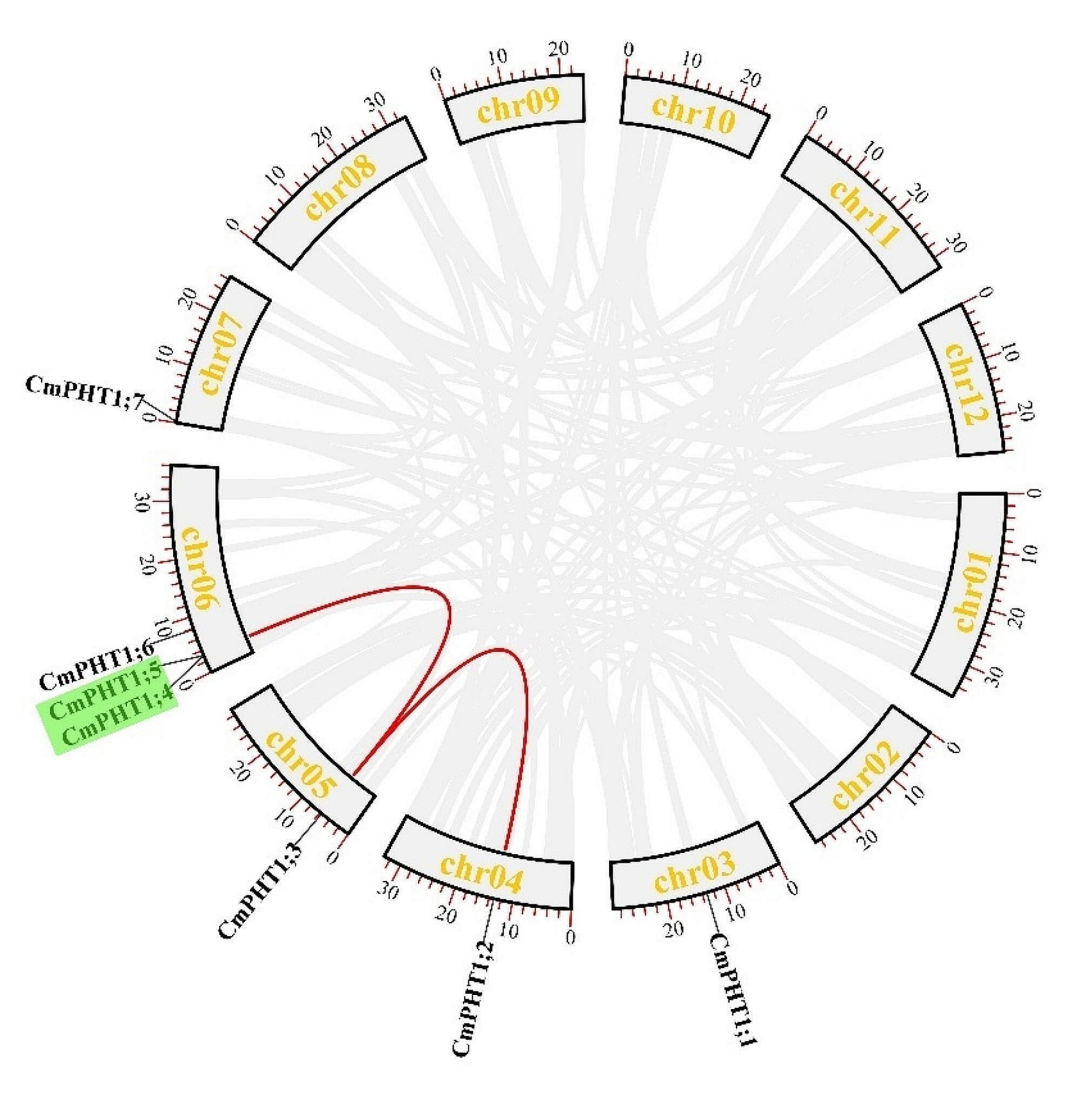
Illustrative depiction of the chromosomal distribution and interchromosomal relationships of *CmPHT1s*. Gray lines, red lines, and green genes indicated all synteny blocks, duplicated *CmPHT1* gene pairs, and tandem duplication of *CmPHT1s* in the melon genome, respectively

The chromosomal location analyses revealed that 7 *CmPHT1s* were unevenly distributed across five chromosomes (chr3, 4, 5, 6, and 7) (Fig. 3). Chromosome 6 had 3 *CmPHT1* genes. There were 2 pairs of segmentally duplicated CmPHT1 genes (*CmPHT1;3-CmPHT1;4 and CmPHT1;3-CmPHT1;2*) and one pair of tandem duplication (*CmPHT1;4-CmPHT1;5*) identified using MCScanX software. Gene distribution and collinearity analysis indicated that the amplification of *CmPHT1* genes in melon occurred mainly through segmentally and tandem duplicated events.

### Cis-elements prediction of CmPHT1 promoters in melon

**Fig. 4:**
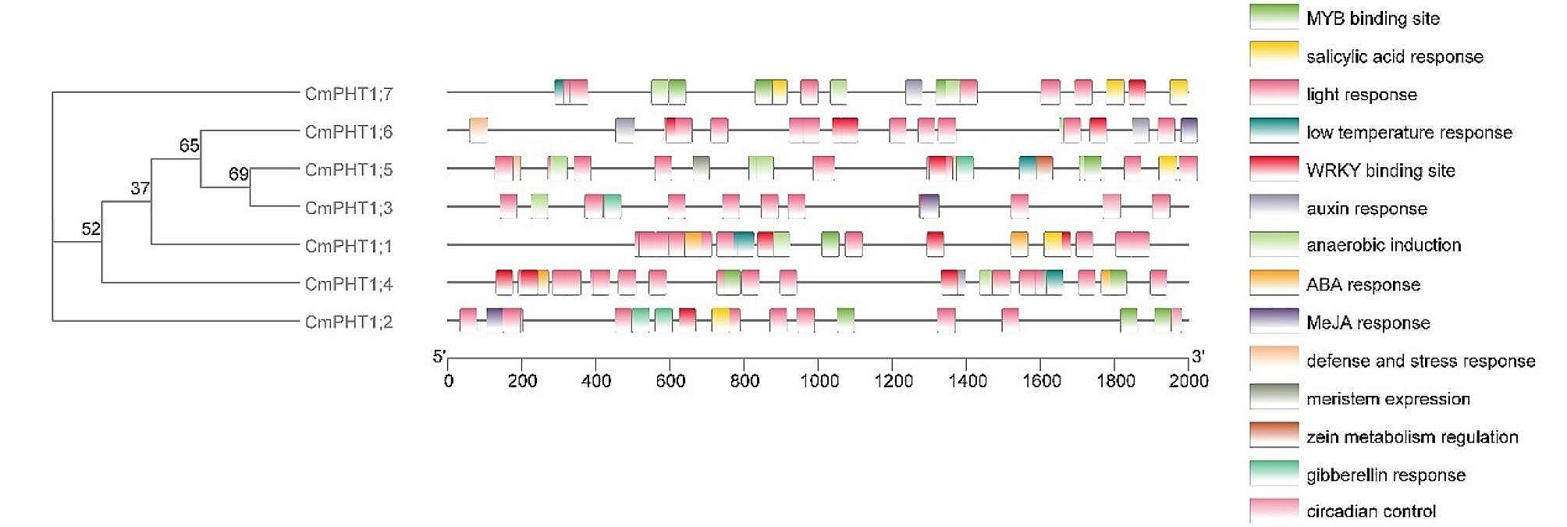
*Cis*-elements in the promoter regions of *CmPHT1s*. The potential *cis*-elements were showed in the 2-kb promoter regions upstream of *CmPHT1* genes, especially the elements related to stress response, plant hormones, WRKY and MYB binding site

Potential cis-elements in the 2-kb promoter regions of *CmPHT1s* were identified using PlantCARE (Fig. 4). The putative MYB transcription factor PHR1 (Phosphate Starvation Response 1)-binding site elements (*P1BS*: GNATATNC) were identified in *CmPHT1;4* promoter (2 sites), *CmPHT1;6* promoter (2 sites), *and CmPHT1;7* promoter (1site). Moreover, *CmPHT1;1*,* CmPHT1;3*,* CmPHT1;5*, and *CmPHT1;7* all had MYB binding sites (MBS and/or MRE). All *CmPHT1* promoters contain one or more WRKY binding sites (W-box: TTGACC). It indicated that *CmPHT1s* might be regulated by MYB and / or WRKY transcription factors. The *cis*-elements responding to hormones such as auxin (TGA-box and AuxRR-core), abscisic acid (ABRE), methyl jasmonate (CGTCA-motif and TGACG-motif), salicylic acid (TCA-element) and gibberellin (P-box, GARE-motif, and TATC-box) exist in *CmPHT1* promoters. *CmPHT1* promoters also contained *cis*-elements responding to abiotic factors, for example, light (TCT-motif, Box 4, TCCC-motif, G-box, and GATA-motif), low temperature (LTR), anaerobic induction (ARE), defense and stress (TC-rich repeats). The *cis*-elements exhibited a special distribution in the *CmPHT1* promoters. For example, there were 4 AREs in the promoter regions of *CmPHT1;5*. The LTR elements were found in *CmPHT1;1*, *CmPHT1;4*,* CmPHT1;5*,* and CmPHT1;7*, indicating that these genes might be responsive to low temperature. In brief, each *CmPHT1* gene possessed its own sets of *cis*-responsive elements. They could be regulated by the different stresses and play a role under the corresponding stress.

### Expression profiling of CmPHT1s with RNA-seq under pathogen infections

**Fig. 5:**
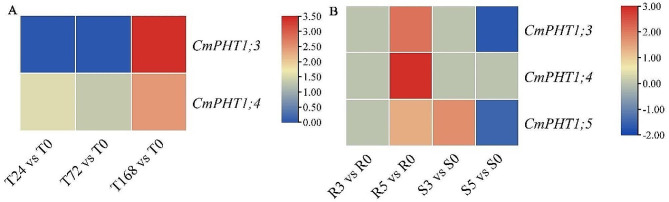
Expression of *CmPHT1s* in melon under the different pathogen infections. The log_2_(foldchange of pathogen infection/CK) values were shown. The genes with thresholds of fold change (FC) ≥ 1.5 and false discovery rate (FDR) < 0.05 were identified as DEGs (same hereinafter). (**A**) the leaf infected by Podosphaera xanthii (Px) in the resistant ‘MR-1’ melon genotypes. T0, T24, T72, and T168 represented the hours post-inoculation. (**B**) the roots infected by Phytophthora capsici in melon. R: resistant cultivar, S: susceptible cultivar. 0, 3, and 5 represented the day post-inoculation

To examine responses of the *CmPHT1* genes to biotic stress, the transcriptome data were extracted for *CmPHT1* gene expression at 0 (CK), 24, 72, and 168 h post-inoculation (hpi) by Podosphaera xanthii (Px) which caused the powdery mildew (PM) in the two contrasting cultivars (the resistant ‘MR-1’ and the susceptible ‘Topmark’) [31], at 0 (control, CK),3 and 5 d post-inoculation (dpi) by Phytophthora capsica in the tolerant line ‘L8’ [32]. *CmPHT1;3* was up-regulated at 168 hpi, while there was no difference at 24 and 72 hpi in resistant leaves (Fig. 5A). *CmPHT1;4* was up-regulated during the whole Px infection in resistant leaves (Fig. 5A). There was no *CmPHT1* gene responding to Px infection in the susceptible melon genotype. It suggested that *CmPHT1;3 and CmPHT1;4* involved in the Pi transport to leaves infected by Px in resistant melon genotype.

The global *CmPHT1s* of the resistant and susceptible genotypes at 3 and 5 dpi compared with CK in roots were analyzed (Fig. 5B). *CmPHT1;3*, *CmPHT1;4*, and *CmPHT1;5* were up-regulated at 5 dpi in the resistant genotype. *CmPHT1;3* and *CmPHT1;5* were down-regulated in the susceptible genotype compared with CK. Phytophthora capsici inhibited the expression of *CmPHT1;3 and CmPHT1;5* in the susceptible genotype roots. It indicated that *CmPHT1;3*,* CmPHT1;4*, and *CmPHT1;5* played a positive role by promoting the uptake of Pi in resistance to P. capsici in the melon root.

### Expression profiling of CmPHT1s with RNA-seq under cadmium stress

**Fig. 6:**
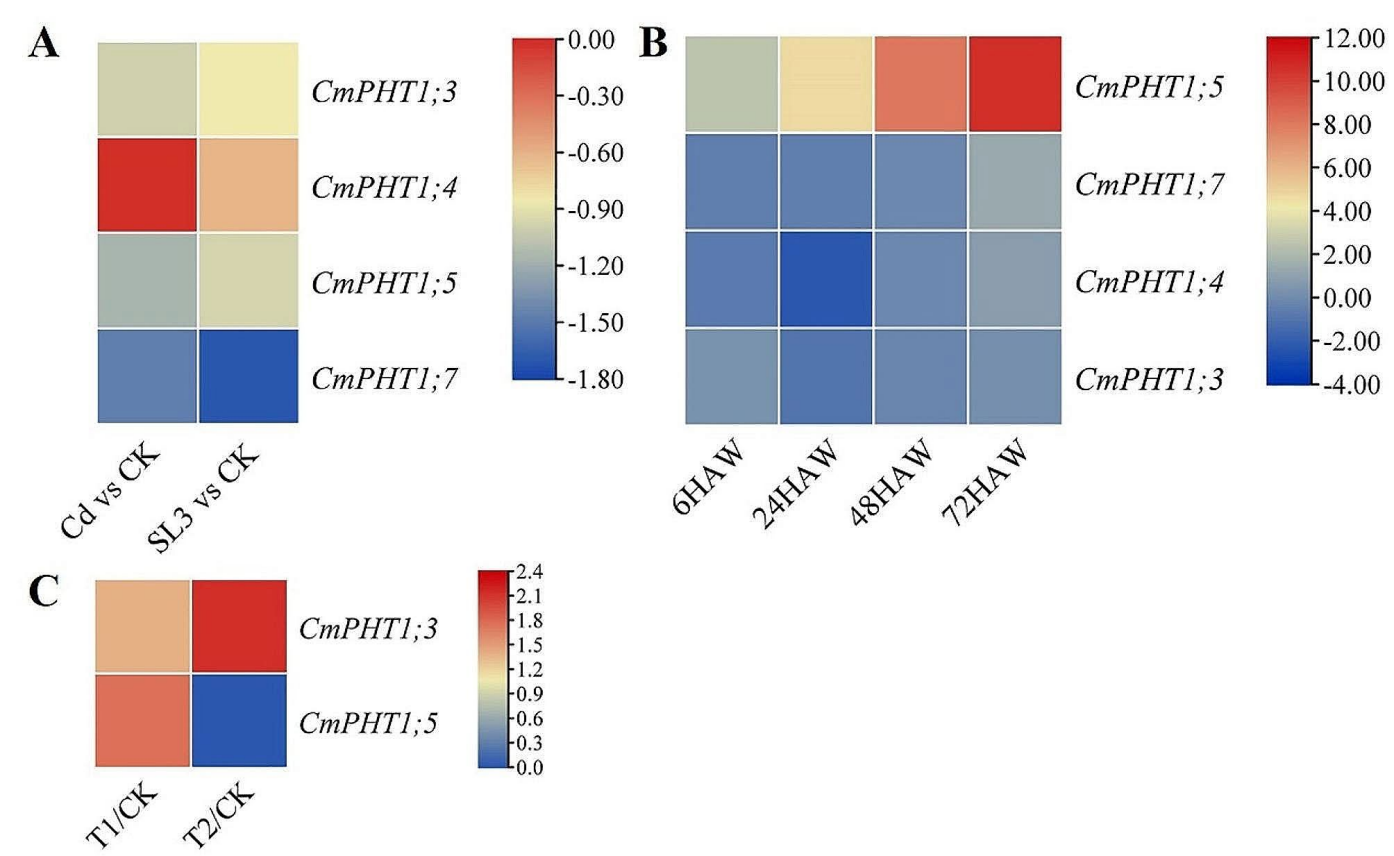
Expression patterns of *CmPHT1s* responding to abiotic stresses. (**A**) cadmium (Cd) stress in melon root. The log_2_(fold change) values were shown. CK: control, Cd:300 µmol L^− 1^ CdCl_2_, SL3: CdCl_2_-stressed seeds pretreated with 1 µmol L^− 1^ GR24 solution. (**B**) waterlogging in melon hypocotyls. The log_2_(fold change) values were shown. HAW: hour after waterlogging. (**C**) the different root-zone CO_2_ concentrations. The log_2_(foldchange of elevated CO_2_ concentrations /CK) values were shown. CK: 0.037% (ambient air), T1:0.5%, T2:1.0%

The transcriptome data under cadmium (Cd) stress in the melon root were analyzed to check *CmPHT1* expression [33]. The expression of *CmPHT1s* in melon root was suppressed by Cd and not improved by pretreated with 1 µmmol L^− 1^ GR24 solution (Fig. 6A). It indicated that Cd inhibited the expression of *CmPHT1s* and the Pi absorption by root from the cultivation medium. It may be one way of growth inhibition for melon seedlings by Cd.

### Expression profiling of *CmPHT1s* with RNA-seq under waterlogging stress

The *CmPHT1s* were identified as participating in the development of adventitious roots induced in melon with the transcriptome profiling data under waterlogging [34]. *CmPHT1;5* was significantly up-regulated during waterlogging, and *CmPHT1;7* was significantly up-regulated at 72 HAW (Fig. 6B). Interestingly, it was corresponding to the AREs in the promoters of *CmPHT1;5* and *CmPHT1;7* (Fig. 4). Waterlogging might induce some factors to bind the ARE elements and activate the expression of *CmPHT1;5* and *CmPHT1;7*.

### Expression profiling of CmPHT1s with RNA-seq under the elevated root-zone CO_2_

Rhizosphere CO_2_ is vital for crop productivity [35]. *CmPHT1;3* and *CmPHT1;5* were up-regulated under 0.5% (T1), while only *CmPHT1;3 was* up-regulated under 1.0% (T2) (Fig. 6C). It indicated that high rhizosphere CO_2_ was favorable to the transcription of *CmPHT1;3* and *CmPHT1;5*. The elevated root-zone CO_2_ caused the anaerobic condition, which induced the expression of *CmPHT1;3* and *CmPHT1;5*.

### Expression profiling of CmPHT1s with RNA-seq under the low-phosphate stress (LP) in two contrasting melon genotypes

**Fig. 7:**
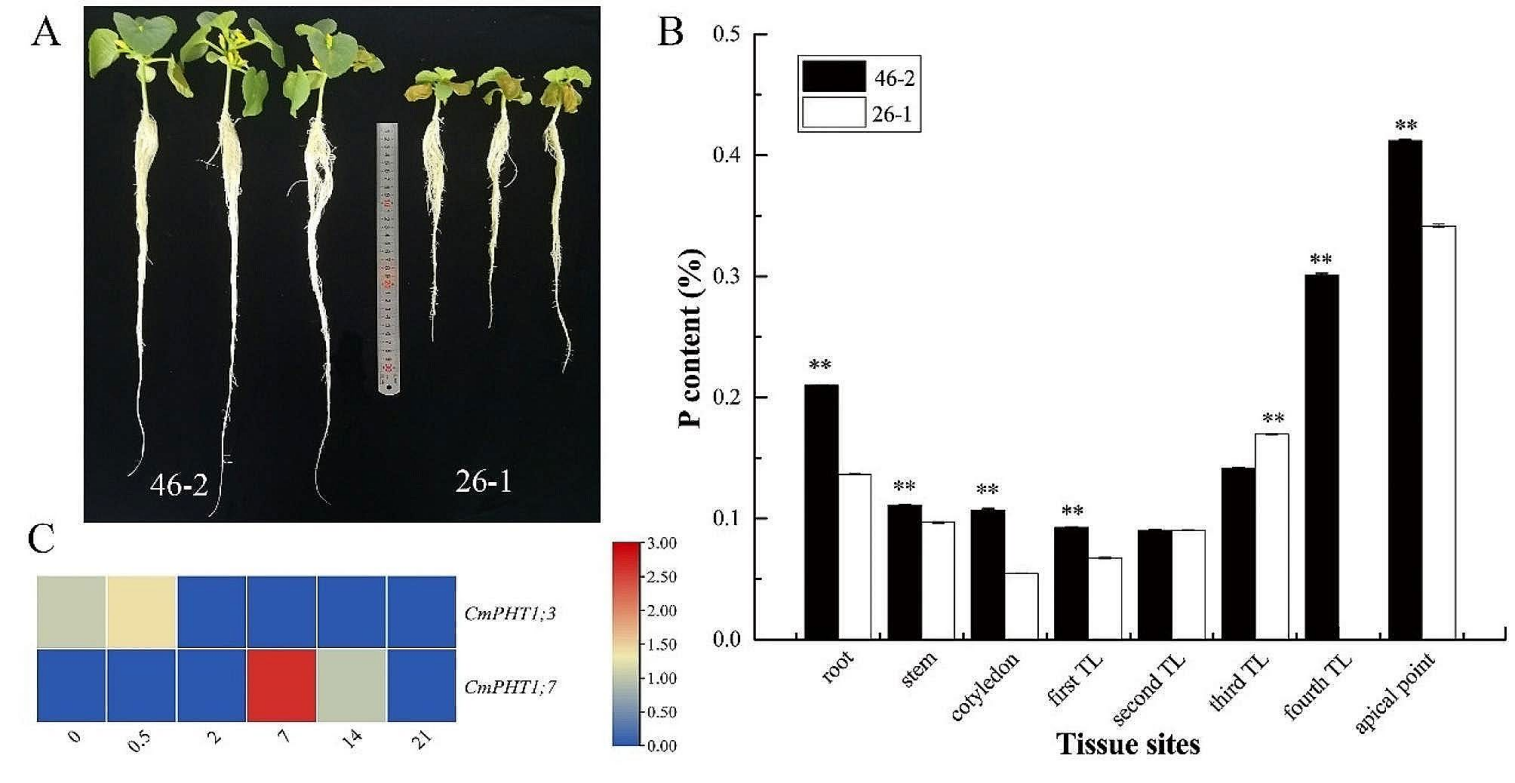
Melon seedlings (**A**), P content (**B**), and expression patterns of *CmPHT1* genes in root (**C**) under low-phosphate stress. ‘46 − 2’ and ‘26 − 1’ were the tolerant and sensitive cultivars to LP, respectively. The log_2_(foldchange of ‘26 − 1’ / ‘46 − 2’) values were shown. 0,0.5,2,7,14, and 21 represented the days of treatment. TL: true leaf

The growth status of the tolerant cultivar ‘46 − 2’ was better than sensitive cultivar ‘26 − 1’ under LP (Fig. 7A). The P contents of root, stem, and leaves, except the second and third leaves in ‘46 − 2’ were significantly higher than ‘26 − 1’ (Fig. 7B). *CmPHT1;3* at 0 and 0.5 d and *CmPHT1;7* at 7 and 14 d after LP were significantly upregulated in the sensitive cultivar compared with the tolerant cultivar. It indicated that *CmPHT1;3* was rapidly induced in the sensitive cultivar (Fig. 7C). No differences in the expression of *CmPHT1;3* were found after 2 d. The roots mobilized the *CmPHT1s* to absorb more P in the LP-sensitive cultivar, which also reflected that the P content was less in the sensitive cultivar. Combined with the cis-elements, LP might induce the expression of PHR1 and then activate *CmPHT1;7*.

### Expression patterns of CmPHT1s under the high-temperature stress

**Fig. 8:**
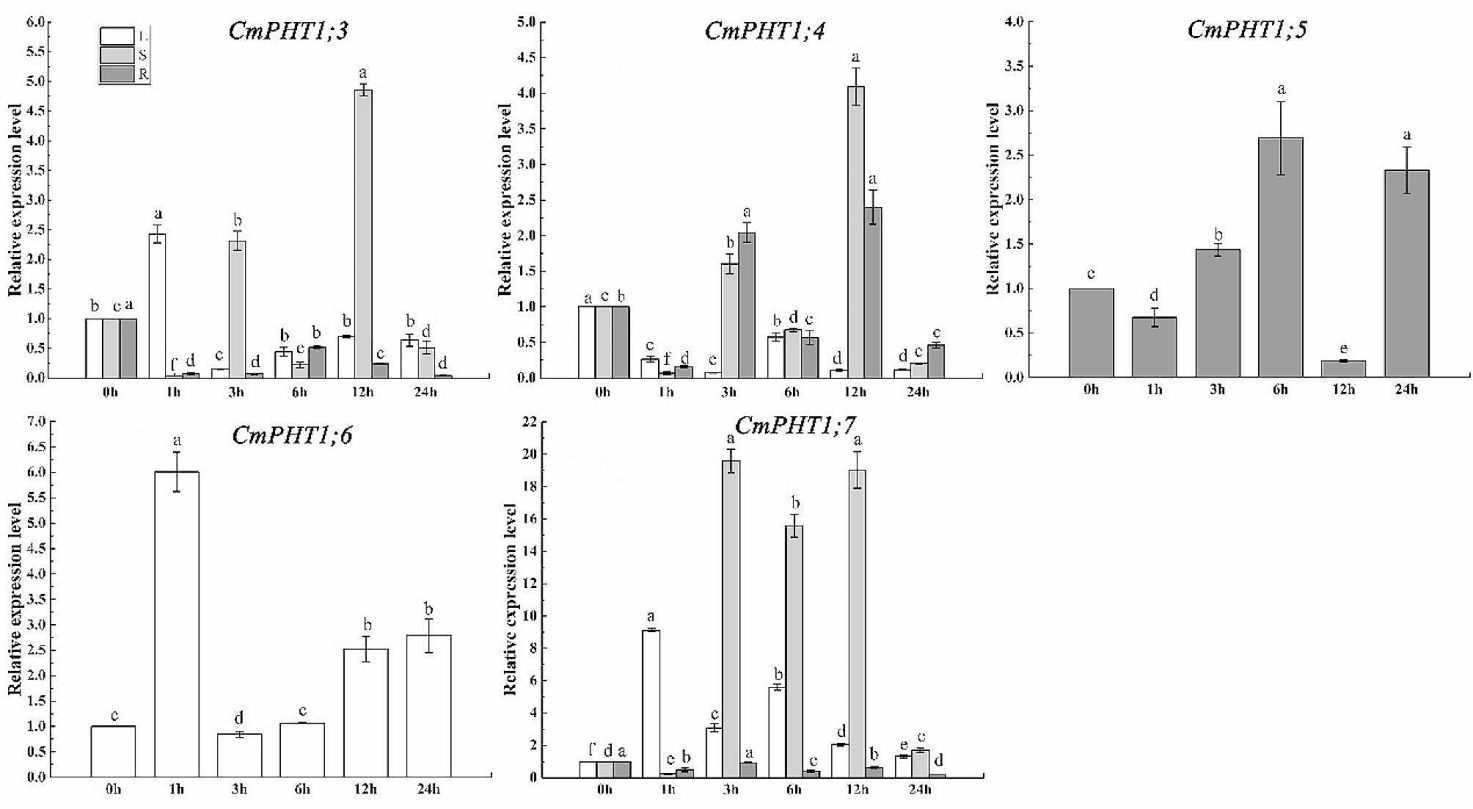
Expression patterns of *CmPHT1s* in melon under high-temperature stress. Significant differences within the same tissue across different treatment times are indicated by lowercase letters (*P* < 0.05). L: leaf; S: stem; R: root

To explore the expression response to high temperature, we measured the transcript level of *CmPHT1s* (Fig. 8). The expression of *CmPHT1;3*, *CmPHT1;6*, and *CmPHT1;7* initially surged, peaking at the 1st h, and subsequently declined in leaves subjected to high-temperature stress (HT) (Fig. 8). *CmPHT1;4* was down-regulated by HT in leaves (Fig. 8), while there was no effect on the transcript level of *CmPHT1;5* in leaves (Fig. 8). The transcript level of *CmPHT1;3*, *CmPHT1;4* and *CmPHT1;7* decreased markedly at 1st h, increased at 3rd h, and peaked at the 12th h in stems. The transcript of *CmPHT1;3* and *CmPHT1;7* in roots decreased under HT. While the expression of *CmPHT1;4* and *CmPHT1;5* decreased firstly at 1st h, and then showed an increasing trend subsequently in roots.

### Expression of CmPHT1s under the low-nitrate stress

**Fig. 9:**
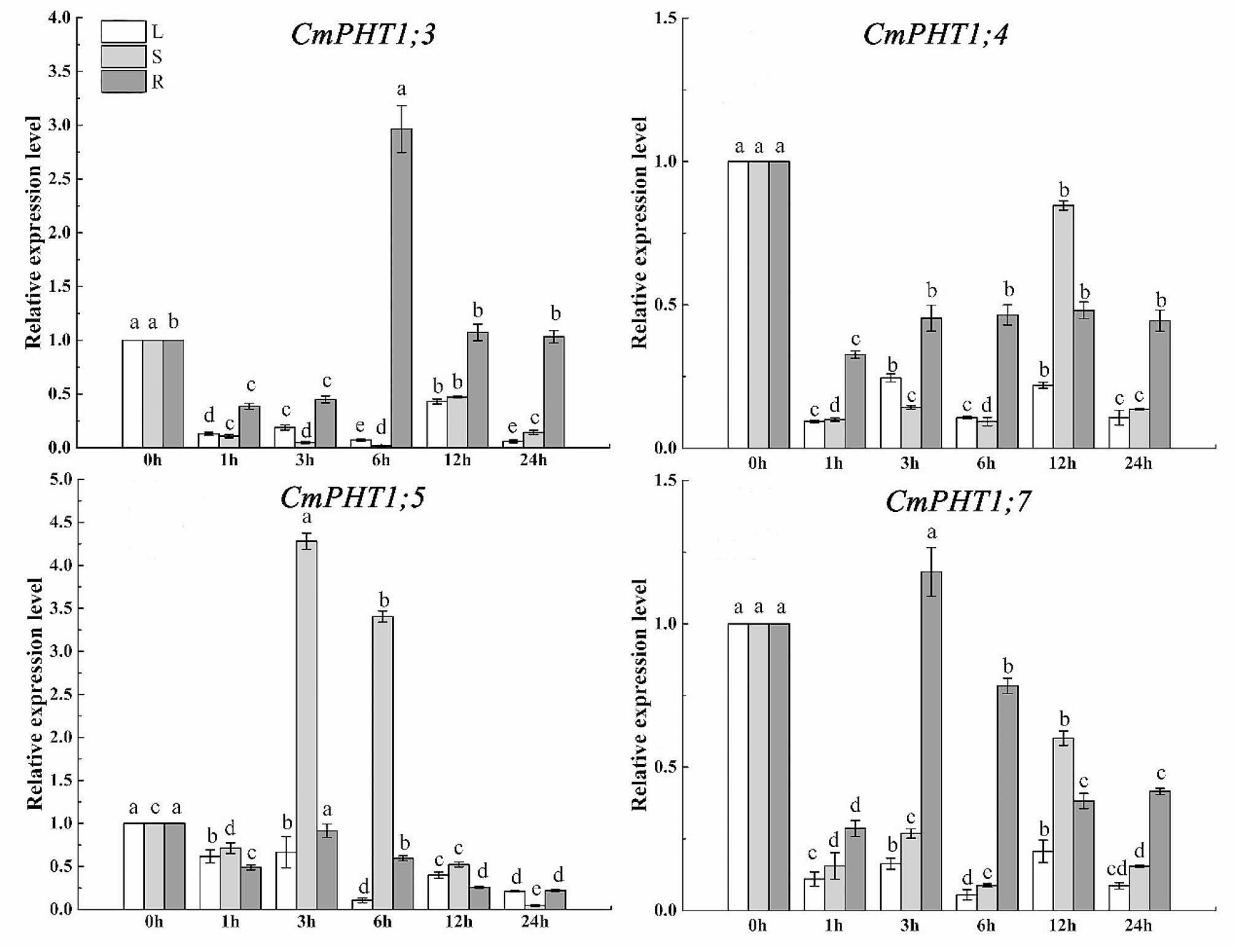
Expression patterns of *CmPHT1s* under the low nitrate stress in melon. Significant differences within the same tissue across different treatment times are indicated by lowercase letters (*P* < 0.05). L: leaf; S: stem; R: root

The *CmPHT1;3* expression was downregulated in leaves and stems under the low-nitrate stress (LN) (Fig. 9). *CmPHT1;3* expression was downregulated at 1st -3rd h and upregulated at 6th h in roots. *CmPHT1;4* was downregulated in leaves, stems, and roots by LN. *CmPHT1;5* was downregulated in leaves and roots by LN, while upregulated at 3rd and 6th h in stems. *CmPHT1;7* was downregulated in leaves, stems, and roots by LN. As a whole, LN suppressed the expression of *CmPHT1s*.

### Expression patterns of CmPHT1s under the low-phosphate stress

**Fig. 10:**
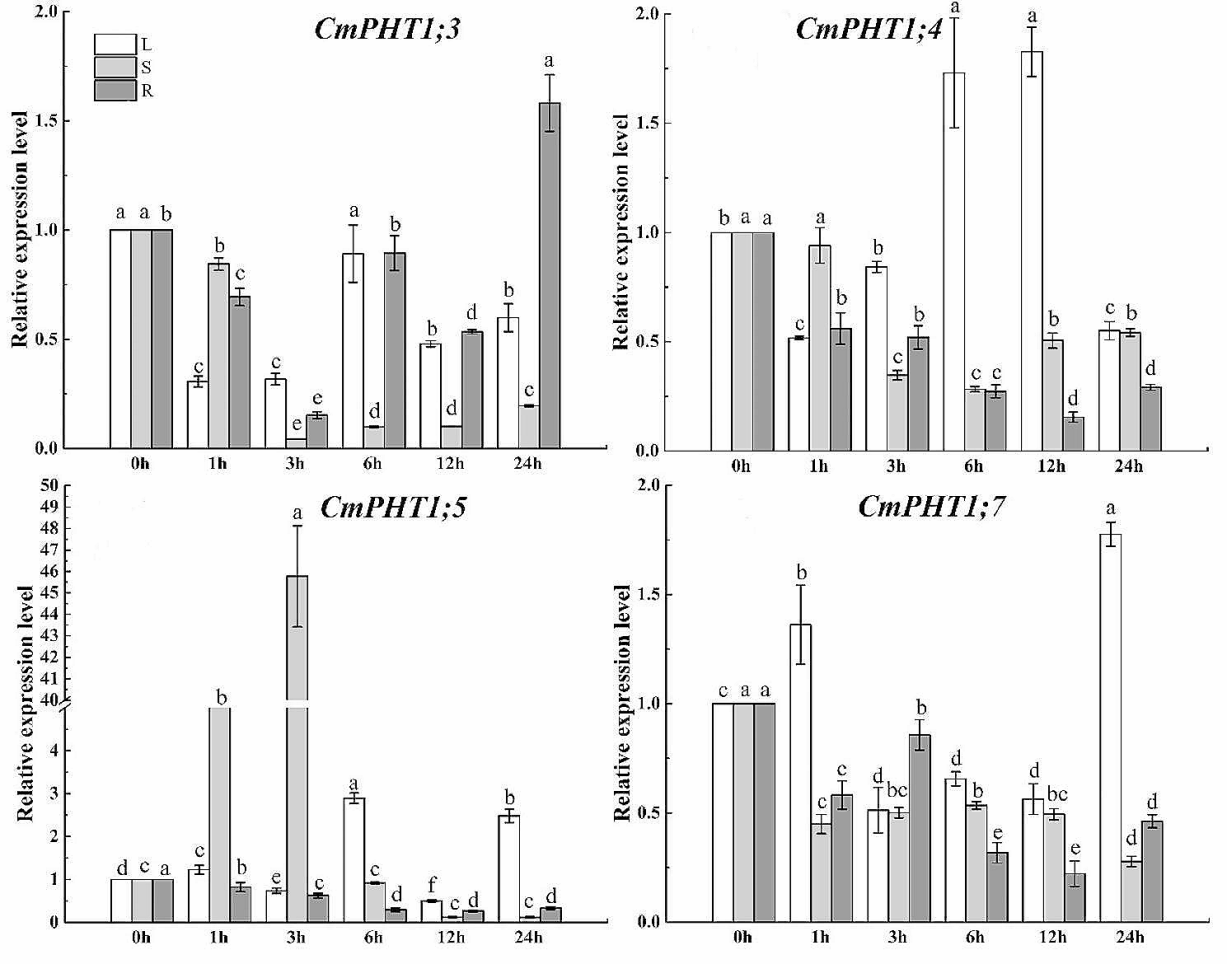
Expression patterns of *CmPHT1s* under the LP stress in melon. Significant differences within the same tissue across different treatment times are indicated by lowercase letters (*P* < 0.05). L: leaf; S: stem; R: root

The *CmPHT1s* expression was assessed in the LP-tolerant cultivar (Fig. 10). *CmPHT1;3* was downregulated in leaves and stems under LP. It was upregulated at 24th h in roots. *CmPHT1;4* was upregulated in leaves at the 6th and 12th h and downregulated in stems and roots by LP. *CmPHT1;5* was upregulated in leaves at the 6th and 24th h, in stems at 1st and 3rd h, and downregulated in roots by LP. *CmPHT1;7* was downregulated in leaves, stems, and roots by LP, except the 1st h and 24th h in leaves.

### CmWRKYs directly bound to the W-box element in CmPHT1;5 promoter

**Fig. 11:**
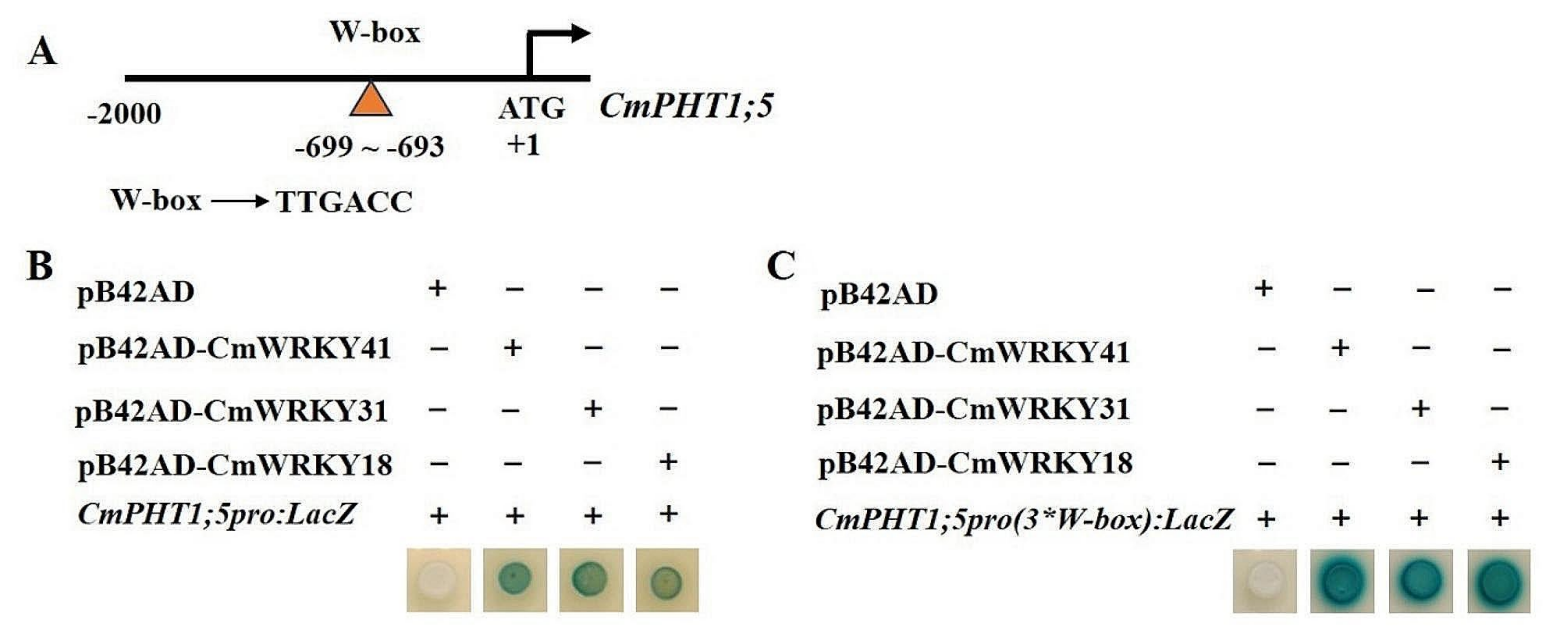
CmWRKYs bind to *CmPHT1;5* promoter. (**A**) Schematic diagram of *CmPHT1;5* promoter. The orange triangle represents the sequence of the W-box element in the *CmPHT1;5* promoter and its relative positions to ATG. The predicted WRKY-binding site is located from − 699 to-693. (**B**) Y1H displays 3 CmWRKYs can interact with *CmPHT1;5* promoter; (**C**) Three tandem copies of W-box element (TTGACC) were synthesized and ligated into pLacZ vector for Y1H assays

*CmPHT1;5* was upregulated, and three *CmWRKY* genes (*CmWRKY31*, *CmWRKY41*, and *CmWRKY18*) were upregulated under stresses by analyzing the RNA-seq data (Table S4). Further, we analyzed the promoter sequences and found that a W-box element existed from − 699 bp to -693 bp in *CmPHT1;5* promoter (Figs. 4 and 11A). It was assumed that CmWRKY31, CmWRKY41, and CmWRKY18 may be the upstream transcription factor (TF) of *CmPHT1;5*. To verify this hypothesis, a yeast-one-hybrid (Y1H) assay was performed with *CmWRKYs*. All yeast cells grew well on SD/-Ura/-Trp medium; however, the yeast strain EGY48 blued only when co-transformed CmWRKY31, CmWRKY41or CmWRKY18 and the *CmPHT1;5* promoters (Fig. 11B). It showed that pB42AD-*CmWRKYs* fusion protein strongly activated the expression of *LacZ*. In addition, we identified that WRKY recognition sites were W-box element (TTGACC) by Y1H (Fig. 11C). The results demonstrated that CmWRKYs are directly bound to the W-box element in *CmPHT1;5* promoter.

## Discussion

In recent years, numerous PHT1 gene families have been discovered in diverse plant species employing comparative genome methodologies. The identification of seven putative *PHT1s* in the melon genome came true through extensive bioinformatics analysis. Studying the gene structure, promoter region cis-elements, evolutionary relationship, chromosomal distribution, and expression profiles of *CmPHT1s* can offer insights into the mechanisms underlying the conservation, expansion, and functional diversity of *PHT1s* across the entire Cucurbitaceae family, facilitating a comprehensive understanding of their potential roles.

### The transcript of CmPHT1s was regulated by abiotic and biotic stresses

*PHT1s* play an important role in Pi absorption and transport [36]. In the present study, *CmPHT1* genes were expressed in melon roots, hypocotyl, stems, and leaves, where they performed functions in Pi uptake and translocation under different stresses. *CmPht1;3*, *CmPht1;4*, *CmPht1;5*, *CmPht1;6*, and *CmPht1;7* were expressed in leaves (Figs. 5, 9 and 10). *CmPht1;3*, *CmPht1;4*, *CmPht1;5*, and *CmPht1;7* were expressed in stems and roots (Figs. 6, 7, 8, 9 and 10). The expression level of *CmPHT1s* was different under different stresses. *CmPht1;4*, *CmPht1;5*, and *CmPht1;7* were downregulated by the short-term (shorter than 24 h) LP stress in the LP-tolerant cultivar roots (Fig. 10C), while *CmPht1;3*, *CmPht1;4*, and *CmPht1;5* were upregulated significantly induced by the long-term LP stress in the roots [29]. The transcripts of *AtPHT1s* are found in both roots and shoots [8, 36–38]. Transcripts of *AtPHT1;6* are most abundant in flowers [8]. Transcripts of all *AtPHT1s* except *AtPHT1;6* accumulate under Pi starvation [39, 40]. *CmPHT1;1* and *AtPHT1;6* were clustered into C3 (Fig. 2). Transcripts of *CmPHT1;1* weren’t detected in melon plants under LP stress. It indicated that *CmPHT1;1* wasn’t induced by LP stress. *AtPHT1;*5 facilitates the movement of Pi between source and sink organs, thereby adjusting phosphate homeostasis [41]. *CmPHT1;5* and *AtPHT1;5* have a close phylogenetic relationship (Fig. 2). Further verification is required to ascertain whether *CmPHT1;5* facilitates the mobilization of phosphate between source and sink organs. *SiPHT1*;*1*, *1*;*2*, *1*;*3*, and *1*;*8* were expressed in shoots of the LP-best-performing genotypes in foxtail millet (*Setaria italica*) [42]. Genotypes exhibiting low phosphate (Pi) contents stimulated the expression of a greater number of *SiPHT1s* [42]. It is consistent with our results that more *CmPHT1s* were induced in the LP-sensitive cultivar (Fig. 7).

*CmPHT1s* were inhibited by LN in melon roots (Fig. 9). It has been reported that N availability regulates Pi-deficiency responses [43]. Under P-deficiency, N supplement is conducive to P acquisition, while N-starvation restrains the P-starvation responses, for example the expression of *PHT1* genes [44]. A downregulation of PSR (Phosphate starvation response) genes emerges in rice and maize under N starvation [45, 46]. Three major signaling factors SPXs, PHR, and PHO2 have been involved in N–P interaction. It has been reported that PHR is positively regulated by N at transcriptional and post-transcriptional levels [47, 48]. However, the stability of PHR1 decreases under N-starvation [49].

High temperature is a common environmental stress that decreases the acquisition of soil Pi by roots. *CmPHT1;3*,* CmPHT1;4*,* CmPHT1;6*,* and CmPHT1;7* were downregulated in melon roots under high temperature (Fig. 8). In Arabidopsis, *AtPHT1;1* and *AtPHT1;2* were significantly downregulated under heat stress [50]. After one hour of heat stress, *PHT1;1*, *PHT1;4*, and *PHT1;6* were downregulated in barley roots [51]. However, *OsPT8* modulates auxin signaling and boosts tolerance to high-temperature conditions in *Nicotiana tabacum* [52].

After pathogen infection, *CmPHT1s* exhibited upregulation in the leaves of the resistant cultivar and downregulation in the leaves of the susceptible cultivar (Fig. 5). It indicated that the pathogen inhibited the expression of *CmPHT1s* in the susceptible cultivar leaves. *CmPHT1;3* and *CmPHT1;4* can participate in the transport of Pi into the leaves. It has been reported that the proportion of the total phosphate in shoots was more in mildewed than in healthy barley [53]. Our previous research found that *CmPHT1s* were upregulated in the powdery mildew (PM)-resistant cultivar compared with the PM-susceptible cultivar under LP-stress (not published). Foliar applications of mono-potassium phosphate fertilizer inhibit powdery mildew development in nectarine trees [54]. The foliar sprays of phosphate and potassium salts can control PM caused by Sphaerotheca fuliginea in cucumber [55]. The *OsPT8*-overexpressed rice plants compromised the resistance against M. oryzae and X. oryzae pv. Oryzae. It indicates that the cross-talk between *OsPT8*, Pi signaling, and plant immunity exists [56]. The excess of Pi enhances disease susceptibility to M. oryzae in rice. It indicated that Pi and defense signals must operate in a coordinated manner to control disease resistance [57]. Even though *CmPHT1s* aren’t the pivotal genes that regulate the defense against the pathogen, they could positively participate in the resistance to PM in melon.

Thus, the transcript of *CmPHT1s* is differentially regulated by stresses. The expression profiles of *PHT1s* could be integral to stress adaptation mechanisms. Nonetheless, the regulatory mechanisms remain to be studied further.

### Cis-elements in CmPHT1 Promoters revealed their functional divergence

The growth and development of melons are susceptible to a variety of biotic and abiotic stresses. The stresses such as pathogen infection, nutrition deficiency, heavy metal pollutants, waterlogging, and extreme temperature often have injurious effects on melon growth and development [31–35]. The *cis*-regulatory elements in the *CmPHT1* promoter regions are the premise of CmPHT1 functions [58]. Most *CmPHT1* promoters contained abiotic stress-responsive elements, suggesting that *CmPHT1* genes widely participate in stress responses. *P1BS* to which PHR1 binds is a conserved motif responding to phosphate deficiency in crops [59]. *CmPHT1;4*,* CmPHT1;6*,* and CmPHT1;7* possess *P1BS* in the promoter regions (Fig. 4). *CmPHT1;6* was not induced by Pi deficiency, although it contained P1BS elements (Figs. 4 and 10). It was consistent with the previous conclusions [60, 61]. There were 4 AREs in the promoter regions of *CmPHT1;5* (Fig. 4). *CmPHT1;5* was significantly upregulated under waterlogging stress (Fig. 6B). It indicated that *CmPHT1;5* participated in the response to waterlogging in melon roots. Each *CmPHT1* gene has plenty of light-responsive cis-elements (Fig. 4). It has reported that red light regulated the transcript of *PHT1;1* by binding two PHYTOCHROME-INTERACTINGFACTORs and ELONGATED HYPOCOTYL 5 to the *PHT1;1* promoter [62]. It suggests that *PHT1s* involve positively in the crop growth and development.

The *cis*-elements, such as methyl jasmonate, gibberellin, salicylic acid, auxin and abscisic acid response, exist in the *CmPHT1* promoters. It implies that *CmPHT1s* are regulated by hormone signals. The regulation of *PHT1s* is affected by phytohormones that mediate stress signals, such as ethylene and abscisic acid, as well as other phytohormones like auxin, cytokinin, and gibberellin [63]. In Arabidopsis, the expression of *AtPHT1;1* was significantly reduced by cytokinin and increased by auxin [64]. The transcript of *Pht1;1* was induced by auxin and GA in maize [65]. Further study on the regulatory mechanism of phytohormones to *CmPHT1s* needs to be done.

### CmWRKYs are the upstream TFs of CmPHT1;5

Of the 7 *CmPHT1s*, *CmPHT1;5* was chosen for the in-depth function analysis based on the following reason: first, *CmPHT1;5 was* widely responsive to stresses (Figs. 5B, 6B and C, 8, 9 and 10), indicating its potential broader functions. Second, based on the bioinformatical analysis of its promoter, we noticed that it contains various cis-regulatory elements associated with stress responsiveness (anaerobic induction, MYB binding site, low-temperature response, WRKY binding site, defense and stress response), growth and development (light response and meristem expression), metabolic regulation (zein metabolism regulation), and hormone responsiveness (salicylic acid response and gibberellin response) (Fig. 4), indicating that it may be regulated not only by stress but also growth and development. Thirdly, *CmPHT1;5* is not only responsible for the absorption of Pi, but also for its transport from root to shoot. All the evidence strongly supports *CmPHT1;5* as a promising candidate gene for in-depth functional characterization in future study.

WRKY TF family members play key roles in regulating plant Pi homeostasis. Based on the transcriptional data published in melon, *CmWRKY41*,* CmWRKY31*,* and CmWRKY18* shared the same expression patterns with *CmPHT1;5* (Table S4). Y1H assay results showed three *CmWRKYs* directly bound to the W-box element in its promoter (Fig. 11). It indicated that *CmWRKYs* are the upstream TFs of *CmPHT1;5*. AtWRKY45 directly upregulates of *AtPHT1;1* expression by binding to two W-boxes within the *AtPHT1;1* promoter under Pi starvation [66]. *OsWRKY21* and *OsWRKY108* function redundantly to promote Pi uptake by activating *OsPHT1;1* expression under Pi-replete condition [67]. *OsWRKY74* significantly influenced Pi acquisition by regulating OsPHT1;3, OsPHT1;4, and OsPHT1;10 transporter proteins in *OsWRKY74-*overexpressed lines [68]. Under the abundant Pi, on one hand, PtoWRKY40 binds to the W-box of *PtoPHT1s* promoter to repress their expression; on the other hand, PtoWRKY40 interacts with PtoPHR1-LIKE3 (PtoPHL3) to prevent PtoPHL3 from binding to the P1BS of *PtoPHT1s* and thus reduced *PtoPHT1s*’ expression. However, Pi deficiency decreased *PtoWRKY40* abundance and therefore initiates *PtoPHT1s*’ expression [69]. These reports indicated that *PHT1s* are the target genes of WRKY TFs under the different Pi conditions. These works provide the ideas and methods for future studies. However, many further works need to be done in melon, for example, *CmPHT1* function, the *CmWRKY-*regulatory mechanism, and the stress-regulatory mechanism to CmPHT1s expression.

*CmPHT1s* played the positive roles in stress tolerance, and some TFs regulated the *CmPHT1*expression. Modern breeding methods, such as transgenesis and gene editing technique, are effective and trusted in improving crop stress tolerance. Previous reports have shown that overexpression of PHT1s or their upstream TFs in crop improved the tolerance to stresses. Overexpression of *OsWRKY74* significantly enhanced tolerance to Pi starvation in rice [68]. *OsPHT1;3* overexpression led to increased Pi concentration in rice by improving the absorption and transport of Pi under extremely LP regimes [12]. *EsPHT1;5* overexpression in salt cress enhanced plant tolerance to LP and salinity by playing an integral role in Pi acquisition and distribution [70]. These findings indicated that *PHT1s* and their upstream TFs can serve as targets for genetic manipulation to improving crop tolerance to stresses. We can transfer *CmPHT1s* or their positive regulatory TFs into melon and obtain the *CmPHT1s* overexpression plants. The plants will improve the Pi absorption and/or transport to meet Pi demand for stress resistance.

This study on *CmPHT1s* has many limitations for lack of function validation. The intensive exploration of their biological function and upstream TF identification should be carried out. For example, *CmPTH1s* are positively or negatively regulated by CmWRKYs, and the crucial function of *CmPTH1s* and their upstream TFs in melon. Moreover, the hormone-regulatory mechanism for *CmPHT1s* expression, for example auxin, abscisic acid, jasmonate, salicylic acid, and gibberellin, should be clarified under the stresses. Therefore, transgenic melon via overexpressing or/and knocking out the *CmPTH1s* and their upstream TF genes should be obtained, their physiologic functions response to various stresses and the regulatory mechanism need to be demonstrated in future research.

## Conclusions

Our work identified 7 *CmPHT1* genes in the melon genome. A comprehensive bioinformatic analysis of the *CmPHT1* gene family was conducted, including basic characteristics, conserved domains, phylogenetic relationships, exon-intron structures, and promoter cis-elements. By combining expression pattern analysis with promoter studies, *CmPHT1s* were shown to respond to a variety of stresses, including phosphate deficiency, heat, anaerobic conditions, and pathogen infection. Interestingly, *CmPHT1;3* and *CmPHT1;5* displayed the most significant upregulation in response to these stresses. Future, CmWRKYs regulated the *CmPHT1;5* expression by binding to the W-box element. In conclusion, the candidate *CmPHT1s* responsive to stresses were screened, laying some foundations for penetrative studies on their functional mechanism in Cucurbitaceae crops.

## Methods

### Plant materials, growth conditions, and stress treatments

#### Low-phosphate stress

To investigate the response of *CmPHT1* genes to LP stress in melon, two contrasting melon genotypes ‘46 − 2’ (LP-tolerant genotype) and ‘26 − 1’ (LP-sensitive genotype) were grown in the greenhouse facilities of Shanghai Jiao Tong University. These two genotypes were collected and saved by our group. The LP treatments were carried out under hydroponic conditions. Standardized seedlings with fully developed first true leaves were transferred to plastic trays, with each tray accommodating 18 plants. These trays were filled with 6 L of half-strength modified Hoagland’s nutrient solution. The dose for LP treatment was 0.001 mM KH_2_PO_4_ and the control (CK) was 0.25 mM KH_2_PO_4_ based on the previous research [71]. The plants were cultured at 28 °C/18 °C day/night with a 14-hour (h) photoperiod, 600 ± 20 µmol m^− 2^ s^− 1^ irradiance, and 50–75% relative humidity. LP treatment lasted for 21 days (d).

#### High-temperature treatment

‘46 − 2’ (LP-tolerant genotype) was used in high-temperature treatment. The high temperature was maintained at 45 °C during the day and 35 °C during the night, with a 14-hour photoperiod, for one day [72]. For control treatments, the temperature was 28℃/18℃ for day and night (14 h/10 h).

#### Low-nitrate treatment

‘46 − 2’ (LP-tolerant genotype) was used in low-nitrate treatment. The nitrate concentration of low-nitrate stress was 1% of control (8 mM) for one day [45]. The culture condition was same with LP stress.

Samples were collected at 0 h, 1 h, 3 h, 6 h,12 h, 24 h, 2 d, 4 d, 7 d, 14 d, and 21 d post-treatment. These samples were harvested to assess various biochemical, physiological, and morphological parameters, with at least three subsamples taken for each measurement.

### Download of data resources

The latest genome sequence and annotation files of melon (DHL92 V4.0) were downloaded from CuGenDB (http://cucurbitgenomics.org/organism/20) to construct a local database. The genome data of Arabidopsis and rice were downloaded from the ensemble database (http://plants.ensembl.org/index.html) to analyze all the candidate *PHT1* genes and construct the phylogenetic tree.

### Genome-wide identification of *CmPHT1s* in melon

To get the comprehensive and accurate identification of *CmPHT1s* in melon genome, the following methods were taken. Firstly, 9 *Arabidopsis* PHT1 proteins (https://www.arabidopsis.org/) and 13 OsPHT1 proteins (https://rapdb.dna.affrc.go.jp/) were queried to search CmPHT1 proteins across the melon genome with ‘Blast Compare Two Seqs (Sets)’ in TBtools software [73]. Secondly, the accuracy of deduced protein sequences was confirmed by searches for homologous sequences deposited in the NCBI database (https://www.ncbi.nlm.nih.gov/cdd/?term=) using the BLAST (Basic Local Alignment Search Tool) with *E*-values < 10^-5^. Thirdly, the candidate protein sequences were obtained and identified using the NCBI Conserved Domain Database (https://www.ncbi.nlm.nih.gov/cdd/) (*E*-value < 10^5^, other parameters set as defaults) and SMART databases (http://smart.embl-heidelberg.de/) to ensure the presence of sugar (and other) transporter domains. The *PHT1* genes that contained the transporter domain and the hits of *E*-values < 10^5^ were considered *CmPHT1* genes [74]. The confirmed *CmPHT1* genes were renamed according to their positions on melon chromosomes.

### Protein property analysis, the subcellular localization, and transmembrane topology prediction of CmPHT1 in melon

The physicochemical characteristics of each CmPHT1 protein were determined using the ExPASy online tool (http://www.expasy.org/tools/). The parameters included the amino acid count, molecular weight (kD), theoretical isoelectric point (pI), atomic composition, grand average of hydropathicity (GRAVY), aliphatic index, extinction coefficient (M^− 1^ cm^− 1^), and instability index. The sub-cellular localization of PHT1 proteins was predicted with ProtComp 9.0 (http://linux1.softberry.com/berry.phtml?topic=protcomppl&group=programs&subgroup=proloc) and Plant-PLoc 2.0 (http://www.csbio.sjtu.edu.cn/bioinf/Cell-PLoc-2/). The transmembrane topology prediction was performed on DeepTMHMM (https://dtu.biolib.com/DeepTMHMM).

### Phylogenetic tree construction

The full-length amino acid sequences of 7 CmPHT1, 9 AtPHT1, and 13 OsPHT1 proteins were made the alignment using ClustalW with default settings in MEGA 7 [75]. An unrooted phylogenetic tree was constructed based on the alignments with the maximum likelihood (ML) method and 1000 bootstraps [76]. For better visualization, the phylogenetic tree was beautified and embellished using the online tool Evolview v2 (https://www.evolgenius.info/evolview).

### Analysis and visualization of gene structure and conserved motifs

The intron-exon distributions of *CmPHT1s* were obtained using GFF annotation files of melon genome. The gene structures of *CmPHT1s* were analyzed and visualized using Graphics of TBtools software [73]. The conserved motifs were identified within the CmPHT1 proteins with MEME online. The ideal breadth of each motif was set to range from 6 to 50 residues. The number of motifs to find was set to 8.

### Collinearity relationship

The genomic sequences and annotation data were scrutinized to extract collinearity data concerning the *CmPHT1s* using the TBtools. Subsequently, the results were imported into the Advanced Circos feature of TBtools for the visualization of expansion patterns within the *CmPHT1* gene family.

### Analysis of cis‑elements in CmPHT1 promoters in melon

The 2 kb upstream sequences of the transcription start site for *CmPHT1s* were retrieved with TBtools [73]. The *cis*-elements were analyzed by PlantCARE (http://bioinformatics.psb.ugent.be/webtools/plantcare/html/) [77]. The cis‑elements were visualized using Graphics-Basic Biosequence View of TBtools software.

### Measurement of P content

The P content (%) was analyzed by inductively coupled plasma atomic emission spectroscopy (ICP 7600, Thermo Fisher Scientific, Waltham, MA, USA) following digestion in a solution comprising 65% (*v*/*v*) HNO_3_ and 72% (*v*/*v*) HClO_4_ (5:1, *v*/*v*) at 220 °C [29].

### Expression patterns of CmPHT1s under diverse stresses

Transcriptome data accessible online were employed to investigate the expression dynamics of *CmPHT1s* in melon under both biotic and abiotic stress conditions [31–35]. The expression data of *CmPHT1s* were searched, analyzed, and visualized.

### qRT‑PCR analysis

The expression patterns of CmPHT1 family genes were analyzed via qRT-PCR. RNA was extracted using a plant RNA isolation reagent (Tiangen Biotech, China) and subjected to reverse transcriptase reactions to synthesize cDNA using PrimeScriptTM RT Master Mix. qRT-PCR was performed on a Roche LightCycler 96 real-time PCR machine (Roche, Basel, Switzerland) with four replicates. The calculation of the expression level was conducted as the relative 2^−△△Ct^ method [78]. Actin was employed as an internal control [29, 72, 79]. The primers are listed in Table S5.

### One-hybrid (Y1H) assay

The genomic DNA was extracted from melon with a Plant Genomic DNA Extract Kit (Beijing Tiangen, China). The *CmPHT1;5* promoter sequence was cloned according to the melon genomic sequence.

For the Y1H assay, pB42AD, CmWRKYs-pB42AD, *CmPHT1* promoters-pLacZ and 3*W-box(TTGACC)-placZ were co-transformed into EGY48, respectively. The both plasmids were confirmed on a SD/-Trp/-Ura plate, and interactions were evaluated on a SD/Gal/Raf/-Trp/-Ura + X-gal plate. Three independent biological replicates were made.

### Statistical analysis

All experimental data were presented as the mean ± SE of at least three biological replicates. ANOVA analysis at *P* < 0.05 was performed to identify significant differences using SPSS Statistics 22.0 (IBM, Chicago, IL, USA). All figures were drawn with TBtools V1.120 (Guangzhou, China) and OriginPro 2022 (OriginLab, Northampton, MA, USA).

### Electronic supplementary material

Below is the link to the electronic supplementary material.

Supplementary Material 1

## Data Availability

All data generated or analysed during this study are included in this published article and its supplementary information files.

## Abbreviations

P: Phosphorus
Pi: Phosphate
P1BS: PHR1-binding site element
BLAST: Basic Local Alignment Search Tool
PI: Isoelectric point
II: Instability index
AI: Aliphatic index
GRAVY: Grand average of hydropathicity
DEGs: Differentially expressed genes
LP: Low-phosphate stress
P1BS: PHR1-binding site element
HT: High-temperature stress
LN: Low-nitrate stress
TL: True leaf
TF: Transcription factor
