# Supplementary material for: Characterization and stress-responsive regulation of CmPHT1 genes involved in phosphate uptake and transport in Melon (Cucumis melo L.)
Source: BMC Plant Biol. 2024 Jul 23;24:696. doi: 10.1186/s12870-024-05405-w (PMC11264433; doi:10.1186/s12870-024-05405-w)
Supplement: Supplementary file 1 — Supplementary Material 1 [file 12870_2024_5405_MOESM1_ESM.docx]

**Supplemental Tables**

Table S1 the basic characteristics of 7 CmPHT1 proteins

| Name | Gene ID | Chr | Number of amino acids | molecular weight /kDa | Pi | atomic composition | extinction coefficient/M^-1^ cm^-1^ | instability index | stability | aliphatic index | grand average of hydropathicity (GRAVY) | sub-cellular localization | Transmembrane Topology Prediction(TMRs) |
| --- | --- | --- | --- | --- | --- | --- | --- | --- | --- | --- | --- | --- | --- |
| CmPHT1;1 | MELO3C011590 | 3 | 533 | 58306.94 | 9.07 | C_2705_H_4141_N_669_O_725_S_21_ | 72685 | 31.32 | stable | 95.16 | 0.395 | Plasma membrane | 12 |
| CmPHT1;2 | MELO3C012861 | 4 | 542 | 59176.86 | 8.6 | C_2736_H_4156_N_676_O_735_S_28_ | 76570 | 28.13 | stable | 88.08 | 0.337 | Plasma membrane | 12 |
| CmPHT1;3 | MELO3C022994 | 5 | 538 | 59371.12 | 8.79 | C_2744_H_4182_N_688_O_733_S_26_ | 78310 | 32.92 | stable | 92.01 | 0.294 | Plasma membrane | 12 |
| CmPHT1;4 | MELO3C006427 | 6 | 519 | 56833.17 | 8.57 | C_2631_H_4013_N_649_O_708_S_24_ | 75205 | 37.02 | stable | 91.21 | 0.393 | Plasma membrane | 12 |
| CmPHT1;5 | MELO3C006428 | 6 | 530 | 57705.39 | 8.93 | C_2668_H_4098_N_662_O_716_S_25_ | 78310 | 30.07 | stable | 94.68 | 0.387 | Plasma membrane | 12 |
| CmPHT1;6 | MELO3C006975 | 6 | 528 | 58452.22 | 8.71 | C_2696_H_4136_N_668_O_728_S_28_ | 67060 | 28.37 | stable | 91.31 | 0.275 | Plasma membrane | 12 |
| CmPHT1;7 | MELO3C017055 | 7 | 556 | 60910.61 | 8.78 | C_2780_H_4264_N_712_O_766_S_31_ | 63300 | 35.85 | stable | 90.23 | 0.351 | Plasma membrane | 12 |

Table S2 CmPHT1s comparison on the amino acid level. BLAST program was used to obtain the percentage of identity (in bold) and similarity

| Identity/ similarity | CmPHT1;1 | CmPHT1;2 | CmPHT1;3 | CmPHT1;4 | CmPHT1;5 | CmPHT1;6 | CmPHT1;7 |
| --- | --- | --- | --- | --- | --- | --- | --- |
| CmPHT1;1 | **100%** | 86% | 84% | 82% | 83% | 75% | 67% |
| CmPHT1;2 | **74%** | **100%** | 85% | 84% | 87% | 75% | 65% |
| CmPHT1;3 | **73%** | **74%** | **100%** | 85% | 89% | 74% | 65% |
| CmPHT1;4 | **70%** | **76%** | **75%** | **100%** | 86% | 78% | 67% |
| CmPHT1;5 | **71%** | **76%** | **80%** | **76%** | **100%** | 77% | 66% |
| CmPHT1;6 | **60%** | **61%** | **63%** | **61%** | **63%** | **100%** | 62% |
| CmPHT1;7 | **52%** | **51%** | **52%** | **52%** | **52%** | **47%** | **100%** |

Table S3 the characteristics of conserved protein motifs, conserved domain, and gene structure of *CmPHT1s*

| Gene | number of conserved motif | conserved domain | number of exon |
| --- | --- | --- | --- |
| CmPHT1;1 | 8 | 2A0109 superfamily | 1 |
| CmPHT1;2 | 8 | 2A0109 | 1 |
| CmPHT1;3 | 8 | 2A0109 | 1 |
| CmPHT1;4 | 7 | 2A0109 | 1 |
| CmPHT1;5 | 8 | 2A0109 superfamily | 2 |
| CmPHT1;6 | 7 | 2A0109 superfamily | 2 |
| CmPHT1;7 | 5 | 2A0109 superfamily | 2 |

Table S4 the Log_2_FC of *CmWRKY* genes under the stresses

| Gene | Log_2_FC | | | |
| --- | --- | --- | --- | --- |
|  | S3 vs S0 | 48 HAW | 72HAW | T1/CK |
| *CmWRKY31* | 3.20 | 3.07 | 3.27 | 1.30 |
| *CmWRKY41* | NA | 1.06 | 2.26 | NA |
| *CmWRKY18* | NA | NA | 1.63 | NA |

Table S5 Sequences of the primers for qRT-PCR

| **Gene ID** | **Gene Description** | **Primer 5**′→**3**′ | **Primer 5**′→**3**′ |
| --- | --- | --- | --- |
| Actin | Reference gene | TCTATTCCAGCCATCTCTC | GACCCTCCAATCCAAAC |
| MELO3C011590 | *CmPHT1;1* | ACCATCATTGGACCGGGAAC | GCAGCCGATATCCCATGACA |
| MELO3C012861 | *CmPHT1;2* | CAACCGAATTGGCTTCGTGG | AGCCAAATGCACCCACCATA |
| MELO3C022994 | *CmPHT1;3* | ATCGTCTCTGCCGCTTTCAA | CAGTGTATCTCGCCGTCTCC |
| MELO3C006427 | *CmPHT1;4* | ATGCCGGAAACTGCTCGTTA | TGAAACCCATGTCGACGGAG |
| MELO3C006428 | *CmPHT1;5* | CCAGTGGGATCAACCGTACC | ACACTTTCGACATGTCCGCT |
| MELO3C006975 | *CmPHT1;6* | CCAACTAGGGTTCGGTCCAC | CCTTTCGTCTCCGTCACCAA |
| MELO3C017055 | *CmPHT1;7* | AGCAGCAACCTCTTCCAGTC | GGCTTGGAGTCTGGCTTCAT |
